# Foodborne Pathogenic Bacteria: Prevalence and Control—Volume I

**DOI:** 10.3390/foods13101531

**Published:** 2024-05-15

**Authors:** Chunlei Shi, Shimo Kang

**Affiliations:** 1 MOST-USDA Joint Research Center for Food Safety, State Key Laboratory of Microbial Metabolism, School of Agriculture and Biology, Shanghai Jiao Tong University, Shanghai 200240, China; 2 CAS Key Laboratory of Nutrition, Metabolism and Food Safety, Shanghai Institute of Nutrition and Health, University of Chinese Academy of Sciences, Chinese Academy of Sciences, Shanghai 200031, China

## 1. Introduction

From the farm to the dining table, foodborne pathogenic bacteria can contaminate food at any stage of the food production, processing, delivery, preparation, and consumption chain, posing a critical threat to the safety of food systems worldwide [1]. *Staphylococcus aureus*, *Escherichia coli*, *Salmonella*, *Listeria monocytogenes*, *Campylobacter*, and *Vibrio parahaemolyticus* are some of the most common foodborne pathogenic bacteria, and food products contaminated by them traverse intricate global trade networks, posing many disease risks to millions of consumers annually [2]. Foodborne diseases can result from unsafe food storage, processing, preservation, and infected workers, as well as several forms of environmental contamination, including pollution in water, soil, air, infected livestock, and animal feces [3]. Some other factors that increase the incidence of foodborne diseases include the adaptation of pathogens to new environments, the formation of biofilms, the acquisition of virulence factors, and the development of antimicrobial resistance in foodborne pathogenic bacteria [4].

To enhance the safety of our food system, the first step is to know how the food system has been, and could still be, contaminated by common pathogenic bacteria, as well as other emerging and re-emerging pathogenic bacteria. On the other hand, we need to know how these bacteria could survive different storage, processing, and preservation processes in the food system. Biofilm formation and antimicrobial resistance could explain the mechanisms of bacterial survival. However, much is unknown. Once basic information is acquired, we can prevent and control the contamination of foodborne pathogenic bacteria to keep us far away from the pathogens’ attacks.

We are pleased to present this Special Issue on “Foodborne Pathogenic Bacteria: Prevalence and Control”, which contains eleven research articles and two review articles on the detection, prevalence, growth, survival and control. In addition, this Special Issue also covered topics related to rapid detection, persistence in food processing environment, antimicrobial resistance, stress adaptation, antibacterial and antibiofilm mechanisms, etc., as alternative and sustainable innovations to prevent and control the contamination of pathogenic bacteria in the food system. We present a brief overview of each contribution.

## 2. Detection and Prevalence of Pathogenic Bacteria in Food Systems

Foodborne pathogenic bacteria contamination in food systems is a serious issue that can lead to numerous diseases and even death. The WHO has estimated that at least 33 million years of healthy life are forfeited due to the consumption of unsafe food globally every year [5]. Hence, the early detection of foodborne pathogenic bacteria is essential to ensure a safe food supply and to prevent foodborne diseases. Jiang et al. (Contribution 1) developed a rapid and visual DNA detection method named “Cas12aVIP” by utilizing the isothermal and trace-DNA amplification features of recombinase polymerase amplification (RPA), the trans-cleavage activity of Cas12a, and the chromatic phenomena of the cationic-conjugated polythiophene derivative PMNT/ssDNA complex under natural light. The Cas12aVIP method is highly specific, and can accurately detect *E. coli* O157:H7 samples without the results being affected by other foodborne microorganisms. The detection can be accomplished in less than 40 min, and the signal is visible to the naked eye under natural light. This method presents various rapid nucleic acid detection applications in food safety.

Antimicrobial resistance has emerged as a widespread threat to the prevention and treatment of bacterial infections. It occurs through spontaneous mutation(s) and the transfer of genetic material, such as transposons, plasmids, etc. [6]. The plasmid-mediated *tet*(X4) gene conferring resistance to tigecycline has become a threat to food safety. Zhang et al. (Contribution 2) evaluated the genomes of over six hundred *tet*(X4)-producing *E. coli* isolates from Asia and Europe in public databases, and their international prevalence and molecular characterization were analyzed in this study. Phylogenomic results indicated that *tet*(X4)-producing *E. coli* isolates fell into seven lineages, and their international spread mainly occurred in Asian countries, especially China, Pakistan, Singapore, and Malaysia. The mobile genetic element ISCR2 might contribute to the spread of *tet*(X4). This study highlights the importance of enhancing monitoring and control of the spread of the *tet*(X4) gene among *E. coli*.

## 3. Growth and Survival of Pathogenic Bacteria in Food Systems

For foodborne pathogenic bacteria to cause illnesses, the first step must be that the bacteria can grow and survive in harsh food environments. Two articles reported the results of *L. monocytogenes* growth potential in different leafy vegetable cultivation conditions and their persistence in ready-to-eat food processing environments, respectively. Culliney and Schmalenberger (Contribution 3) investigated the influence of vegetable species and variety, cultivation method, and seasonality of cultivation on the growth of *L. monocytogenes* on leafy vegetables. The results showed that plant species and variety influenced *L. monocytogenes* growth potentials. A significant seasonality effect was found between batches. Liu et al. (Contribution 4) identified four *L. monocytogens* STs (ST5, ST121, ST120, and ST2) in two read-to-eat (RTE) food plants between 2019 and 2020 in Shanghai, China. The biofilm-forming ability of the four ST isolates was related to their different growth stages. Moreover, ST5 and ST121 showed greater adaptability to stressful environments, and were able to survive sub-lethal concentrations of chlorine sanitizers. Hence, ST5 and ST121 should be paid more attention, and stronger surveillance in food processing plants in Shanghai is required.

The survival of pathogenic bacteria in the food chain and adaptation to adverse environments are relevant to their capacities of biofilm formation, stress adaptation, virulence, and antibiotic resistance, which were covered by three research articles. Ejaz et al. (Contribution 5) evaluated heavy metal tolerance, antimicrobial resistance (AMR), and biofilm formation of isolated bacteria from dairy and non-dairy products. The results showed that the majority of isolated bacteria and most of the isolated bacteria with heavy metal tolerance showed strong biofilm-forming traits. AMR was associated with stronger biofilm producers. Moreover, the danger of heavy metal tolerance was more serious than AMR. Therefore, the spread of highly drug-resistant biofilm-producing bacteria should be prevented during food storage, processing, and packaging. Sun et al. (Contribution 6) investigated the influence of *ptsH* deletion on stress adaptation and virulence in *C. sakazakii*. The results showed that Δ*ptsH* mutant significantly declined the resistance of biofilm-forming and adhesive, heat stress, and simulated gastric juices, while the superoxide dismutase (SOD) activity, osmotic resistance, and oxidative resistance were increased. RNA-seq method further revealed the possible regulatory mechanism associated with the sulfur metabolism pathway. The results contributed to a further understanding of the pathogenicity of *C. sakazakii*. Biofilm formation of *Salmonella enteritidis* under sublethal ethanol stress was assessed by He et al. (Contribution 7) The results showed that *S. enteritidis* increased quorum sensing and auto-aggregation, and declined the swimming motility under sublethal ethanol stress that enhanced the biofilm formation.

## 4. Promising Control Strategies for Foodborne Pathogenic Bacteria

The pathogenic bacteria that colonize and form biofilms in food systems significantly contribute to the persistent contamination of food products [7]. Biofilms play a crucial role in facilitating bacterial survival and enhancing their virulence. The occurrence of bacterial resistance makes the conventional treatment of biofilms increasingly inefficient. Therefore, novel preventive strategies are needed to control the growth, adhesion, virulence and biofilm formation of pathogenic bacteria in food systems. Four papers reported the results of novel antibacterial agents against planktonic bacteria and biofilms. Su et al. (Contribution 8) present a study focusing on the antibacterial mechanism of linalool on *Shigella sonnei* and evaluate the effect of linalool on the sensory quality of lettuce. The study indicated that linalool is effective *S. sonnei* by damaging cell membrane integrity, increasing reactive oxygen species (ROS) and membrane lipid oxidation, and declining ATP content. Sensory evaluation showed that linalool. Hou et al. (Contribution 9) evaluated the relationship between the regulation of GlpQ degradation of wall teichoic acid (WTA) by lactobionic acid and *S. aureus* biofilm formation. The results suggest that *glpQ* is induced in *S. aureus* to function in WTA degradation with the addition of lactobionic acid, resulting in decreased WTA content and subsequent reduction in adhesion and biofilm formation. Li et al. (Contribution 10) evaluated the effect of enzymes complexed with proteinase K, lipase, and cellulase against *V. parahaemolyticus* biofilm formation. The results demonstrated that the combined enzyme against *V. parahaemolyticus* inhibited the aggregation and the adhesion of biofilm, declining the content and related gene expression of exopolysaccharide. Dong et al. (Contribution 11) investigated the effect of fructooligosaccharides (FOS) and *Lactiplantibacillus plantarum* combined on the growth, adhesion, invasion, and virulence of gene expressions of *L. monocytogenes*. The results showed that *L. plantarum* combined with FOS significantly inhibited the growth of *L. monocytogenes* and reduced the invasion rates to Caco-2 and BeWo cells and virulence-related gene expression of *L. monocytogenes*.

Finally, this Special Issue includes two review articles. A review article by Zhang et al. (Contribution 12) summarized the latest research on viable but non-culturable foodborne pathogenic bacteria, including induction conditions, detection methods, the mechanism of VBNC formation, and possible control strategies. Sithole et al. (Contribution 13) discussed the characteristics of food safety risks related to peanut butter and the effectiveness of the initiatives that are aimed at minimizing these risks.

As our food increasingly comes from all over the world, ensuring food safety becomes more challenging. Hence, we sincerely hope this Special Issue will inspire and guide further research regarding changes in the prevention and control of the contamination of foodborne pathogenic bacteria.

## Data Availability

The data that support the findings of this paper are the articles published in this aforementioned Special Issue. These data were derived from the following available resources: https://www.mdpi.com/journal/foods/special_issues/foodborne_pathogenic (accessed on 10 March 2023).
